# 753. Guideline Adherence Predicts Outcomes of Candidemia in Europe: Results from the ECMM Candida III Multinational European study

**DOI:** 10.1093/ofid/ofac492.038

**Published:** 2022-12-15

**Authors:** Martin Hoenigl, Matthias Egger, Jon Salmanton-Garcia, Philipp Koehler, Jean-Pierre Gangneux, Maiken C Arendrup, Tihana Bicanic, Sevtap Arikan-Akdagli, Oliver A Cornely, ECMM Study Group

**Affiliations:** Medical University of Graz, Department of Internal Medicine, Division of Infectious Diseases, Graz, Steiermark, Austria; Medical University of Graz, Department of Internal Medicine, Division of Infectious Diseases, Graz, Steiermark, Austria; University Hospital Cologne, Cologne, Germany, Cologne, Nordrhein-Westfalen, Germany; University Hospital Cologne, Cologne, Germany, Cologne, Nordrhein-Westfalen, Germany; CHU de Rennes, Rennes, France, Rennes, Bretagne, France; Statens Serum Institut, Copenhagen, Hovedstaden, Denmark; St Georges University Hospital, London, United Kingdom, London, England, United Kingdom; Hacettepe University, Infectious Diseases and Clinical Microbiology, Ankara, Turkey, Ankara, Ankara, Turkey; University of Cologne, Faculty of Medicine and University Hospital Cologne, Cologne, Nordrhein-Westfalen, Germany; European Confederation of Medical Mycology, Basel, Basel-Stadt, Switzerland

## Abstract

**Background:**

The European Confederation of Medical Mycology (ECMM) collected data on epidemiology, risk factors, treatment, and outcomes of culture proven candidemia across Europe in order to assess how adherence to guideline recommendations correlate with outcomes.

**Methods:**

Each participating hospital (number of eligible hospitals per country determined by population size) included the first ∼10 culture proven IC cases after 01-Jul-18 and entered data into the ECMM Candida III database on the FungiScope™ platform. EQUAL Candida Scores (10.1111/myc.12746) reflecting adherence to recommendations of IDSA and ESCMID Guidelines were assessed.

**Results:**

A total of 632 Candidemia cases were included from 64 institutions in 20 European countries. Patients characteristics are displayed in Table 1. Overall mortality was 45% (286/632), and hospital stay was prolonged (median 2 days) for completion of parenteral therapy only in 16% (100/621) of patients. EQUAL Candida Score was evaluable for 589 cases with candidemia (Figure 1). Candida scores correlated significantly with duration of hospitalization (r= 0.442; p< 0.001) and - after exclusion of patients hospitalized < 7 days (n=119) - were significantly higher in patients who survived versus those who died (p< 0.001). Duration of hospitalization was in median 16 days after diagnosis of candidemia. Initial echinocandin treatment was associated a.) with lower overall mortality (42%, 148/353) versus those without initial echinocandin therapy (53%, 126/236; p=0.007), and b.) with longer duration of hospitalization among survivors (median 24 days, IQR 15–40 days vs. median 16 days, IQR 7–33 days; p< 0.001). In those where candidemia was treated for at least 14 days, 78% (239/306) survived, compared to 66% (67/102) in those treated for less than 14 days, but who survived beyond day 14 after diagnosis.

**Conclusion:**

Initial echinocandin treatment was associated with increased overall survival, but also longer duration of hospitalization (hospitalization was prolonged only for completing treatment in 16%). Overall mortality of IC was 45%. EQUAL Candida scores were significantly higher on those who survived, indicating that adherence to clinical guidelines may increase survival.

**Disclosures:**

**Martin Hoenigl, n/a**, Astellas: Grant/Research Support|Gilead: Grant/Research Support|NIH: Grant/Research Support|Pfizer: Grant/Research Support|Scynexis: Grant/Research Support **Maiken C. Arendrup, DMSci, PhD, MD**, Chiesi, Gilead: Honoraria|F2G, Cidara, Scynexis: Grant/Research Support **Oliver A. Cornely, Prof. Dr.**, Abbott: Honoraria|Abbvie: Advisor/Consultant|Actelion: Board Member|Al-Jazeera Pharmaceuticals: Honoraria|Allecra Therapeutics: Board Member|Amplyx: Advisor/Consultant|Amplyx: Grant/Research Support|Astellas: Honoraria|Basilea: Advisor/Consultant|Basilea: Grant/Research Support|Biocon: Advisor/Consultant|Biosys: Advisor/Consultant|BMBF: Grant/Research Support|Cidara: Advisor/Consultant|Cidara: Board Member|Cidara: Expert Testimony|Cidara: Grant/Research Support|CoRe Consulting: Stocks/Bonds|Da Volterra: Advisor/Consultant|DLR: Grant/Research Support|DZIF: Grant/Research Support|Entasis: Board Member|EU Directorate-General for Resarch and Innovation: Grant/Research Support|F2G: Grant/Research Support|German Patent and Trade Mark Office: German patent (DE 10 2021 113 007.7)|Gilead: Advisor/Consultant|Gilead: Grant/Research Support|Grupo Biotoscana/United Medical/Knight: Honoraria|Hikma: Honoraria|IQVIA: Board Member|Janssen: Board Member|Matinas: Advisor/Consultant|Matinas: Grant/Research Support|MedPace: Advisor/Consultant|MedPace: Grant/Research Support|MedScape: Honoraria|MedUpdate: Honoraria|Menarini: Advisor/Consultant|Merck/MSD: Grant/Research Support|Merck/MSD: Honoraria|Molecular Partners: Advisor/Consultant|MSG-ERC: Advisor/Consultant|Mundipharma: Grant/Research Support|Mylan: Honoraria|Noxxon: Advisor/Consultant|Octapharma: Advisor/Consultant|Octapharma: Grant/Research Support|Paratek: Board Member|Pardes: Advisor/Consultant|Pfizer: Grant/Research Support|Pfizer: Honoraria|Projektträger Jülich: Grant/Research Support|PSI: Advisor/Consultant|PSI: Board Member|Pulmocide: Board Member|Scynexis: Advisor/Consultant|Scynexis: Grant/Research Support|Seres: Advisor/Consultant|Shionogi: Board Member|Wiley (Blackwell): Editor-in-Chief, Mycoses.

**Table 1.**
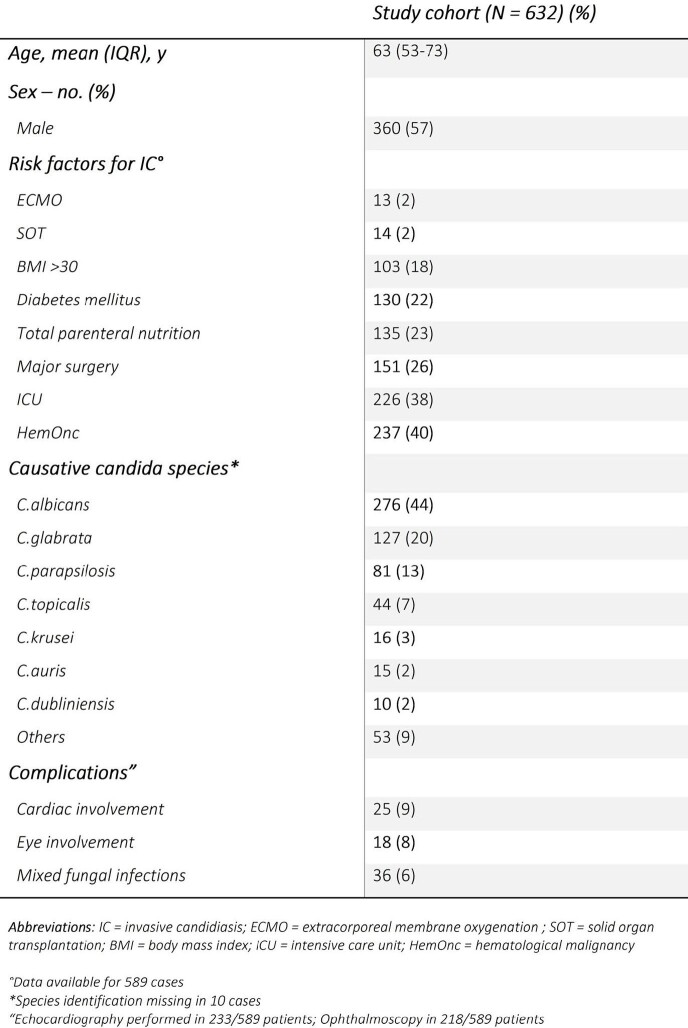
Patient Characteristics and distribution of Candida species

**Figure 1.**
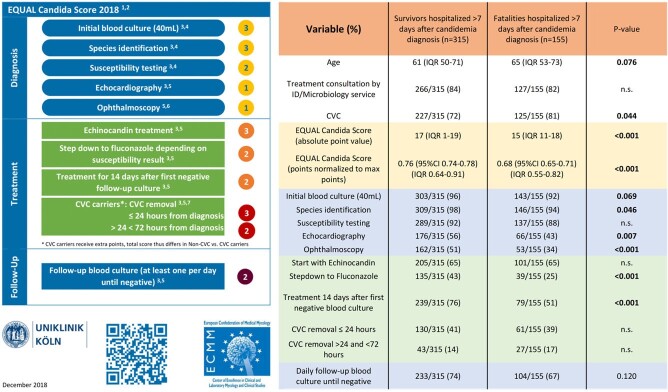
EQUAL Candida Score Card (left) and EQUAL Candida Scores, EQUAL Candida Score variables and demographic data in survivors and non-survivors who were diagnosed >7 days after candidemia diagnosis.

Abbreviations: CVC = central venous catheter; ID = infectious diseases

